# Extraction and In Vitro Skincare Effect Assessment of Polysaccharides Extract from the Roots of *Abelmoschus manihot* (L.)

**DOI:** 10.3390/molecules29092109

**Published:** 2024-05-02

**Authors:** Junjie Wang, Enhui Liao, Zixuan Ren, Qiong Wang, Zenglai Xu, Shufang Wu, Chaoguang Yu, Yunlong Yin

**Affiliations:** 1 Jiangsu Key Laboratory for the Research and Utilization of Plant Resources, Institute of Botany, Jiangsu Province and Chinese Academy of Sciences (Nanjing Botanical Garden Mem. Sun Yat-Sen), Nanjing 210014, China; jjwang@jib.ac.cn (J.W.); leh0406@163.com (E.L.); rzx961838706@gmail.com (Z.R.); xuzenglai@jib.ac.cn (Z.X.); yucg168@sina.com (C.Y.); 2 College of Light Industry and Food Engineering, Nanjing Forestry University, Nanjing 210037, China; shufangwu@njfu.edu.cn

**Keywords:** *Abelmoschus manihot* (L.) root extract, polysaccharides, ultrasound-assisted extraction, skincare effect, hyaluronidase inhibition, elastase inhibition

## Abstract

Obtaining high-added value compounds from agricultural waste receives increasing attention, as it can both improve resource utilization efficiency and reduce waste generation. In this study, polysaccharides are extracted from the discarded roots of *Abelmoschus manihot* (L.) by the high-efficiency ultrasound-assisted extraction (UAE). The optimized condition was determined as solid–liquid ratio SL ratio = 1:20, temperature T = 30 °C and time T = 40 min, achieving an extraction yield of 13.41%. Composition analysis revealed that glucose (Glc, 44.65%), rhamnose (Rha, 26.30%), galacturonic acid (GalA, 12.50%) and galactose (Gal, 9.86%) are the major monosaccharides of the extract. The extract showed a low degree of esterification (DE) value of 40.95%, and its Fourier-transform infrared (FT−IR) spectrum exhibited several characteristic peaks of polysaccharides. Inspired by the wide cosmetic applications of polysaccharides, the skincare effect of the extract was evaluated via the moisture retention, total phenolic content (TPC) quantification, 2,2-Diphenyl-1-picrylhydrazyl (DPPH)-free radical scavenging activity, anti-hyaluronidase and anti-elastase activity experiments. The extract solutions demonstrated a 48 h moisture retention rate of 10.75%, which is superior to that of commercially available moisturizer hyaluronic acid (HA). Moreover, both the TPC value of 16.16 mg GAE/g (dw) and DPPH-free radical scavenging activity of 89.20% at the concentration of 2 mg/mL indicated the strong anti-oxidant properties of the extract. Furthermore, the anti-hyaluronidase activity and moderate anti-elastase activity were determined as 72.16% and 42.02%, respectively. In general, in vitro skincare effect experiments suggest moisturizing, anti-oxidant, anti-radical and anti-aging activities of the *A. manihot* root extract, indicating its potential applications in the cosmetic industry.

## 1. Introduction

In order to seek a balance between economic growth and environmental protection, sustainable chemistry has been proposed [1], which focuses on developing chemical processes and products that are economically viable and environmentally friendly [2]. Sustainable chemistry strives to use renewable resources and minimize waste and pollution [3]. Therefore, obtaining chemical products from natural plants attracts much attention, as it meets the requirement of sustainable chemistry [4,5]. However, the utilization of plant resources is inevitably accompanied with the generation of agricultural wastes. Although these wastes usually can be decomposed naturally without causing serious pollution to the environment, discarding the plant materials is also a waste of resources [6,7]. Therefore, reusing the agricultural wastes to produce high-added value products is important for improving the utilization efficiency of plant resources and reducing wastes.

*Abelmoschus manihot* (L.) is a flowering plant belonging to the *Malvaceae* family, which is widely distributed through eastern Europe and the temperate and subtropical regions of Asia [8,9]. In China, the flowers of *A. manihot* have been used for centuries as folk medicine for the treatment of inflammatory renal/hepatic injuries, and promote tissue repair in ulcers and burns [10,11,12]. Moreover, *A. manihot* flowers are also listed as herbal medicines in the Pharmacopoeia of the People’s Republic of China (2020 edition), permitting their clinical applications in treating kidney diseases like chronic glomerulonephritis and diabetic nephropathy [13]. Phytochemical studies reveal *A. manihot* flowers contain various flavonoids, which are responsible for their pharmacological effects including hepatoprotection [11], neuroprotection [14], anti-oxidation [15], anti-inflammation [16] etc. In addition to their pharmaceutical applications, *A. manihot* leaves are also regarded as a healthy green vegetable as they are rich in micronutrients like iron, folate, β-carotene, etc. [17]. Although the leaves and flowers of *A. manihot* have been intensively investigated and utilized, the roots are often discarded or burned as agricultural wastes, which undoubtedly causes environmental pollution and resource waste. Therefore, obtaining the high-added value products from the roots of *A. manihot* is crucial for the full utilization of *A. manihot*.

Polysaccharides are large molecules constituted by more than ten monosaccharide residues, which exhibit various biological activities including anti-oxidation [18,19], anti-virus [20], immunomodulation [21], and thus have wide applications in skincare [22], drug delivery [23], tissue engineering, [24] etc. Previous studies exhibited that *A. manihot* would be a potential source of bioactive polysaccharides. For example, Zheng et al. obtained the polysaccharide AMPS-a from the flowers of *A. manihot* via a water extraction and alcohol precipitation method followed by column purification [25]. Importantly, AMPS-a showed potent in vitro inhibitory effects on the proliferation of hepatic and gastric cancer cells. Later, Pan et al. also isolated one neutral and two acidic polysaccharides from the stems and leaves of *A. manihot* [26]. An in vitro study indicated that acidic polysaccharides demonstrated higher immunomodulatory activity than the neutral polysaccharide. Nevertheless, to the best of our knowledge, the extraction and bioactivity assessment of the polysaccharides from the *A. manihot* roots remains unexplored.

Motivated by reusing the discarded roots to produce the useful polysaccharides and seeking the potential applications of the polysaccharides, this work develops an ultrasound-assisted extraction (UAE) method to obtain the polysaccharide extract from the *A. manihot* roots and further evaluates its skincare effects via in vitro bioactivity research. UAE is selected as a high-efficient method because the ultrasonic wave cavitation can effectively break the cell walls and accelerate the dissolution of polysaccharides in extractant [27]. In vitro bioactivity assessments demonstrate that the *A. manihot* root extract possesses moisturizing, anti-free radical, hyaluronidase inhibitory and elastase inhibitory activities, indicating the *A. manihot* root extract as a potential anti-oxidant and anti-aging agent in cosmetic products.

## 2. Results and Discussion

### 2.1. Proximate Composition of the A. manihot Root

The proximate analyses including moisture, ash, lipid, protein, total dietary fiber (TDF) and carbohydrates are listed in Table 1. Given the lack of prior reports on the proximate composition of *A. manihot* root, the composition of *A. manihot* leaves serves as a comparison. *A. manihot* leaves showed contents of 13.83% ash content, 27.14% protein and 41.68% carbohydrates [28], while the roots exhibit a higher ash content (18.34 ± 0.45%) and lower protein (10.31 ± 0.68%) and carbohydrates content (7.86 ± 0.61%) than the leaves (Table 1). On one hand, roots primally absorb minerals from the soil, thus the inorganic substances, which are the major components of ash, are commonly higher in roots than in leaves [29]. On the other hand, proteins and carbohydrates are highly involved in various functions in leaves, such as photosynthesis and enzyme activity, hence their content in leaves is usually higher than in roots. Moreover, it is well known that plants in the *Abelmoschus* family (e.g., *Abelmoschus Esculentus* [okra]) are rich in dietary fiber [30]. Therefore, high TDF contents (55.13 ± 0.03%) were also found in the *A. manihot* root, indicating its great potential as a polysaccharide source.

### 2.2. Optimization of Extraction Conditions for A. manihot Root

To improve the extraction efficiency, UAE was employed, as UAE can effectively break the cell walls and accelerate the dissolution of polysaccharides in extractant. In addition, polysaccharides are soluble in H_2_O but insoluble in organic solvents, so H_2_O was selected as the extractant. Three key factors, namely solid–liquid ratio (SL ratio), temperature and time, that significantly influence the extraction yield were mainly investigated. The top three levels (Table 2) for each factor were designed to perform the orthogonal L_9_(3^3^) test for condition optimization and the results are listed in Table 3.

The results demonstrated the order affecting the extraction yields is SL ratio, temperature and extraction time. Based on range analysis, the optimized conditions were determined as an SL ratio of 1:20, a temperature of 30 °C and an extraction time of 40 min, achieving an optimized extraction yield of 13.41 ± 0.24%. The polysaccharide content in the obtained extract was determined as 82.6 ± 0.62% using the phenol-sulfuric acid method. With the optimized condition on hand, we further conducted the chemical characterization and biological activity studies on the extracts.

### 2.3. Characterization of the Extract

#### 2.3.1. Monosaccharide Characterization

**Figure 1:**
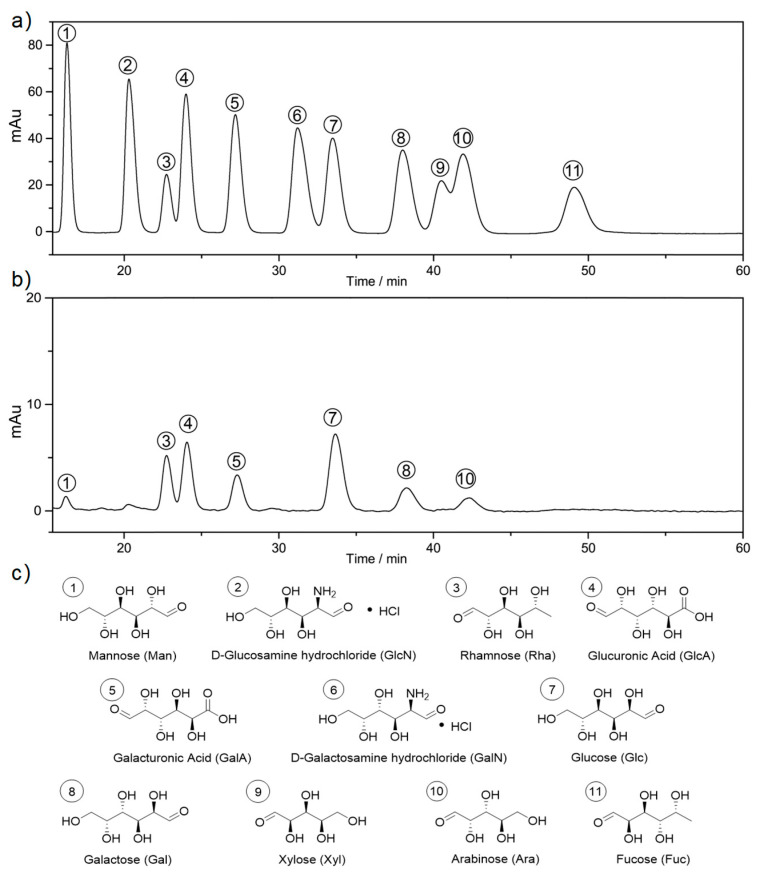
(**a**) HPLC trace of mixed monosaccharide standards; (**b**) HPLC trace of the hydrolyzed polysaccharides mixture; (**c**) name and structures of monosaccharide standards.

The quantitative and qualitative determination of monosaccharides (Figure 1a,b) indicated that the polysaccharides extract mainly consisted of glucose (Glc, 44.65%), rhamnose (Rha, 26.30%), galacturonic acid (GalA, 12.50%) and galactose (Gal, 9.86%). While other monosaccharides including glucuronic acid (GlcA, 2.85%), arabinose (Ara, 2.34%) and mannose (Man, 1.50%) were also detected as hydrolysis products (for structures, see Figure 1c). Similar components were also found in previous literature. For example, Shi et al. [31] reported Glc (50.00%), Rha (21.86%), GalA (16.67%) and Gal (7.40%) are the major monosaccharide compositions of the polysaccharides from *A. manihot*. Furthermore, Pan et al. [26] analyzed a neutral polysaccharide (SLAMP-a) and two acidic polysaccharides (SLAMP-c and SLAMP-d) obtained from the crude polysaccharides. The results revealed that SLAMP-a was mainly composed of Glc with a few Man, Gal and Ara, while the monosaccharide components of SLAMP-c and SLAMP-d were GlcA, Rha, Glc and Gal, in order of amounts. The similarity in monosaccharide composition suggests that the polysaccharide extract from the *A. manihot* root may possess the similar functional properties to those reported, such as anti-oxidant and immunomodulatory effects [26,31].

#### 2.3.2. Degree of Esterification

The degree of esterification (DE), composed of degree of methoxyl and degree of acetylation, is an important characteristic of polysaccharides extract. DE > 50% is defined as high methoxyl (HM), while DE ≤ 50% is regarded as low methoxyl (LM). The extract has a DE value determined as 40.95 ± 0.53% and is therefore classified as LM, which aligns with the previous literature [32].

DE is highly correlated with the gelling and thickening properties of the extract [33]. HM materials are stabilized by the hydrophobic interactions and tend to form gels at a low pH (<4.0) or in the presence of small amounts of sugars (e.g., sucrose). In contrast, LM gel requires divalent metal ions to form the electrostatic and ionic bonds between GalA and carboxyl groups, thereby forming the stable electrostatically gel networks [33].

#### 2.3.3. Infrared Spectroscopy Analysis

**Figure 2:**
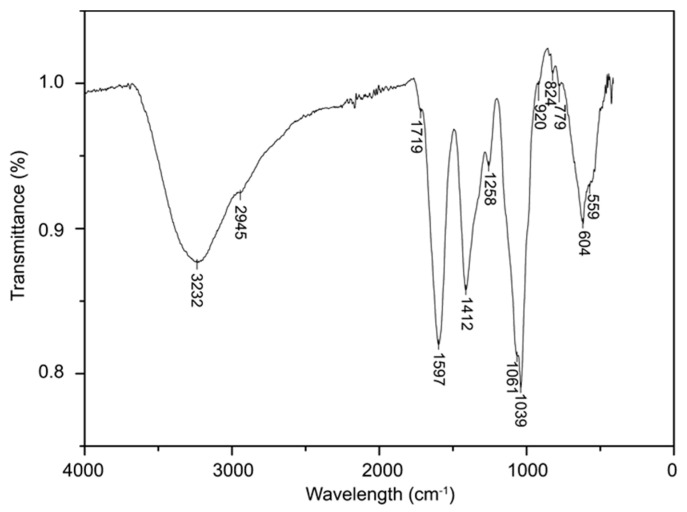
FT−IR spectrum of the polysaccharide extract from *A. manihot* root.

Fourier-transform infrared (FT−IR) spectrum was used to give an insight into the structural properties of the polysaccharide extract (Figure 2). Specifically, the spectrum shows an intense and broad band peak at 3232 cm^−1^, which can be attributed to the O–H stretching frequency of hydroxyl groups. The peak at 2945 cm^−1^ is assigned to C–H absorption, which includes the –CH–, –CH_2_– and –CH_3_ stretching vibration. Moreover, the peak at 1719 cm^−1^ corresponds to C=O stretching vibration of the esterified carboxyl group, while the intense peak at 1597 cm^−1^ represents C=O asymmetrical stretching vibration of the carboxyl group from GalA and GlcA. In addition, the absorption peak of the free carboxyl group COO^−^ was also observed at 1412 cm^−1^. The carbohydrates fingerprint is in the region between 1200 and 800 cm^−1^, where the peaks at 1016 cm^−1^ and 1039 cm^−1^ are attributed to Gal and Glc, respectively. Peaks in the region between 900 cm^−1^ and 400 cm^−1^ are typical vibrations resulting from monosaccharides and oligosaccharides. 

### 2.4. In Vitro Skincare Effect Experiments

#### 2.4.1. Moisture Retention Effect

**Figure 3:**
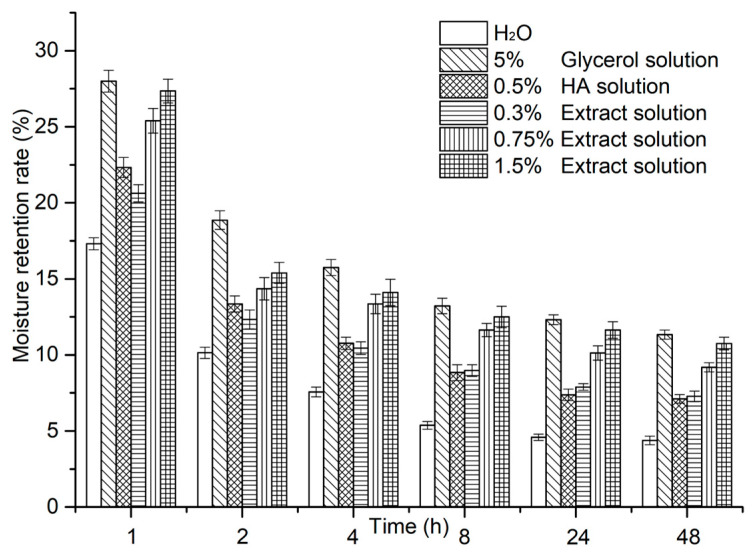
Moisture retention rates of extract solutions with different concentrations and reference (H_2_O, 5% (*w*/*v*) glycerol solution and 0.5% (*w*/*v*) HA solution) at different time points. The moisture retention rates are expressed as mean ± standard deviation of 3 replicates.

Moisturizing is the ability to hydrate by reducing the loss of H_2_O from the skin surface, which is critical for improving the skin barrier function, metabolism and appearance. Polysaccharides not only contain abundant hydrophilic groups (e.g., hydroxyl groups, uronic acid groups), but they also possess huge net-like structures which can prevent water loss. Therefore, polysaccharides are believed to have moisturizing effects. Moisture retention rates were introduced to evaluate the moisturizing effects of *A. manihot* root extract (Figure 3). For comparison, the commercially available moisturizing agents hyaluronic acid (HA) and glycerol were used as references. The result shows that the polysaccharides extract solution (1.5% *w*/*v*) has a 48 h moisture retention rate up to 10.75 ± 0.42%, which is much higher than that of the 0.5% HA solution (7.11 ± 0.29%) but slightly lower than that of 5% glycerol solution. Furthermore, the moisture retention rates were found to be highly concentration dependent, i.e., extract solutions with higher concentrations tend to exhibit increased moisture retention rates. In general, 0.75% and 1.5% extract solutions demonstrate superior moisture retention rates than 0.5% HA solution at all time points, while the moisture retention rate of 0.3% extract solution exceeds that of the 0.5% HA solution after 8 h. Although the moisture retention rates of the tested extract solutions are slightly lower than those of 5% glycerol solution, the extract is still considered as a better moisturizing agent when taking concentration and amount into consideration.

#### 2.4.2. Total Phenolic Content Quantification and DPPH-Free Radical Scavenging Activity

It is known that ultraviolet (UV) radiation from sunlight is one of the main exogenous factors causing skin aging. The UV radiation penetrates the skin and triggers the formation of reactive oxygen species (ROS) and free radicals. These substances can damage the cell structure and function, resulting in skin aging problems such as wrinkles, fine lines, etc. Therefore, the anti-oxidant and free radical scavenging activities of the extract are crucial to its anti-aging effect. 

Phenolic compounds are secondary metabolites in plants, which act as anti-oxidants to trap ROS and free radicals generated during metabolism. Thus, total phenolic content (TPC) is introduced to evaluate the anti-oxidant ability of the extract. The TPC of the extract was determined as 16.16 ± 0.35 mg GAE/g (dw), which is comparable to previous literature [34] reporting TPC values between 12.760 ± 0.486 mg GAE/g (dw) to 30.290 ± 1.873 mg GAE/g (dw), depending on the *A. manihot* harvest time. In addition, it is worth noting that UAE may degrade phenolic compounds and result in lower TPC compared with other extraction methods like batching or microwave assisted extraction [35]. 

**Figure 4:**
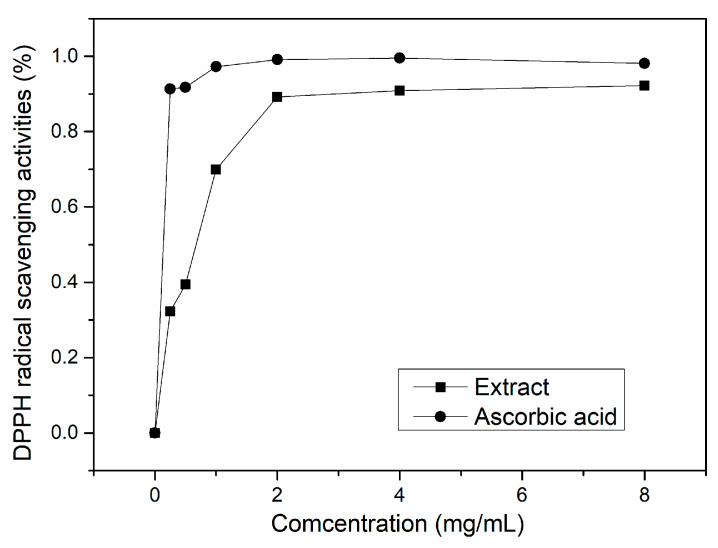
DPPH anti-radical activities of the extract and reference (ascorbic acid).

Besides trapping ROS, anti-oxidants can also neutralize free radicals by donating electrons. The DPPH radical scavenging activity assay was further performed to evaluate the anti-radical ability of the extract. The results indicate a significant enhancement in anti-radical activity as the extract concentration increases when the concentration is below 2 mg/mL (Figure 4). However, further increasing the concentration from 2 mg/mL to 8 mg/mL only leads to a slight improvement in anti-radical activity from 89.20% to 92.24%. Nevertheless, the polysaccharide extract from *A. manihot* root exhibits good free radical scavenging activity, which may be attributed to the high content of Rha, Gal and GalA [34].

#### 2.4.3. Hyaluronidase Inhibition and Elastase Inhibition Activities

The extracellular matrix (ECM) is a complex and highly organized scaffold providing a structural framework for skin growth and flexibility [36]. The ECM is composed of various proteins and polysaccharides such as HA and elastin, which play essential roles in tissue development, maintenance and repair. As the predominant polysaccharide in skin, HA exhibits diverse functions including retaining skin moisture, forming protective barriers and improving skin flexibility. Elastin endows skin with elasticity and resilience when subjected to stretching or deformation. However, the overactivation of hyaluronidase and elastase lead to the breakdown or disorganization of HA and elastin, respectively. This degradation of essential components in the ECM significantly contributes to skin aging [36]. Therefore, inhibition of hyaluronidase and elastase is a promising strategy to protect ECM and prevent skin aging.

The enzyme inhibition experiment demonstrates that the anti-hyaluronidase and anti-elastase activities of *A. manihot* root extract were determined as 72.16 ± 1.79% and 42.02 ± 1.84%, respectively. The extract exhibits a comparable anti-hyaluronidase activity to that of the positive reference dipotassium glycyrrhizate (DPG, 79.91 ± 1.49%), while its anti-elastase activity is significantly lower than that of the positive reference epigallocatechin gallate (EGCG, 87.83 ± 1.59%, Table 4). Nevertheless, the strong anti-hyaluronidase activity and moderate anti-elastase activity make the *A. manihot* root extract a promising candidate as an anti-aging agent.

## 3. Materials and Methods

### 3.1. Materials

The air-dried roots of *A. manihot* were provided by Suzhong Pharmaceutical Group and crushed into a coarse powder using a pulverizer machine before usage. The plant samples were identified by Professor Zenglai Xu as *Abelmoschus manihot* (L.) and were preserved in the herbarium of Nanjing Botanical Garden Mem. Sun Yat-Sen with a voucher No. 2023001. The obtained powder was stored in sealing bags and placed in the dark to avoid the influence of moisture and light. Folin-Ciocâlteu reagents and 2,2-Diphenyl-1-picrylhydrazyl (DPPH) were purchased from Yuanye (Shanghai, China) and Macklin (Shanghai, China), respectively. Spectroscopically pure solvents and commercial standards for HPLC analysis were purchased from Merck (Darmstadt, Germany). Ultrapure water was obtained from a Master-S UP laboratory water purification system (HHitech, Shanghai, China).

### 3.2. Proximate Composition of the A. manihot Root

The proximate composition of *A. manihot* root was determined based on the AOAC official method and the National Standards of the Republic of China. All the analyses were performed in triplicate. Moisture was determined based on the weight loss of a 50 g sample after being dried in the air oven at 105 °C. The ash was measured using a gravimetric method. Namely, 2.0 g dry sample was weighted in crucible and heated in a muffle furnace (FO811, Yamato Scientific Co., Ltd., Tokyo, Japan) at 550 °C for 6 h. The ash of the sample was precisely weighed after cooling down in a glass desiccator. Lipids were extracted and measured by Soxhlet method using petroleum ether as solvent. Proteins were calculated according to the Dumas method, where the values were estimated through multiplying the conversion factor of 6.25 by the nitrogen contents obtained from a CHNS analyzer (UNICUBE, Elementar Americas Inc., Ronkonkoma, NY, USA). Total dietary fiber (TDF) amounts were obtained by enzymatic-gravimetric method, where the pretreated samples were successively digested by α-amylase, protease and amylglucosidase. After precipitation by ethanol, the ash and protein amounts of the filtered precipitate were measured following the same methods mentioned above, and TDF amounts were determined as the weight difference between mass of precipitates and the total mass of ash and protein. Carbohydrates were determined by the difference.

### 3.3. Polysaccharides Extraction

General method: 100 g *A. manihot* root powder (40–60 meshes) was extracted by H_2_O under specific conditions. An ultrasonic machine (KQ-300DA, Kunshan ultrasonic instrument Co., Ltd., Suzhou China) equipped with a digital thermometer was applied to assist extraction under 300 W power intensity and 40 kHz frequency. After the extraction, the sample was centrifuged with a centrifuge (5430R, Eppendorf GmbH, Hamburg, Germany) at 6000 rpm for 5 min and filtered under reduced pressure. Then the filtrate was treated by Sevag reagent for three times to remove the protein. The resulting solution was frozen and dried using a freeze-drying machine (FD-1A-50, Biocool instrument Co., Ltd., Beijing, China) to generate the desired polysaccharides extract. The extraction yield *Y* was calculated according to the following equation:

$$Y\left(\%\right)=\frac{m_{E}}{m}\times 100\%$$

where *m_E_* is the weight of polysaccharides extract (g) and *m* is the weight of the plant sample.

Condition optimization: Three key factors influence the extraction yield *Y* were studied, including solid–liquid ratio (1:10–1:20), temperature (20 °C–40 °C) and time (20 min–60 min). Orthogonal experiments L_9_(3^3^) under designed conditions were performed and the range analysis was conducted to achieve the optimum condition.

The polysaccharide content was measured using the phenol-sulfuric acid method based on the calibration curve of glucose solutions.

### 3.4. Characterization of the Extract

#### 3.4.1. Monosaccharide Composition

Monomeric profile analysis of polysaccharides was conducted based on the pre-column 1-phenyl-3-methyl-5-pyrazolone (PMP) derivatization method. Specifically, 5 mg polysaccharides extracts were hydrolyzed in 1 mL trifluoroacetic acid solution (2 M) at 121 °C for 2 h. The samples were subsequently dried under nitrogen flow, washed by 3 mL methanol (MeOH) and dissolved in 5 mL H_2_O. Then, a serial dilution (10 μg/mL–500 μg/mL) of mixed monosaccharide standard solutions was prepared (rhamnose (Rha), arabinose (Ara), galactose (Gal), glucose (Glc), xylose (Xyl), mannose (Man), galacturonic acid (GalA), glucuronic acid (GlcA), galactosamine hydrochloride (GalN), glucosamine hydrochloride (GlcN)). Afterwards, 0.2 mL standard solutions or samples were dissolved in 0.2 mL sodium hydroxide solution (NaOH, 0.5 M) and reacted with 0.5 mL PMP solution (0.5 M) at 70 °C for 1 h. The reaction mixtures were then neutralized by hydrochloride (HCl) solution, washed by chloroform and diluted to 0.5 mL for chromatographic analysis. High-performance liquid chromatography (HPLC, Ultimate 3000, Thermo Scientific Inc., Waltham, MA, USA) was performed with a ZORBAX EclipseXDB-C_18_ column. A phosphate buffer (pH 6.8) and acetonitrile (MeCN) was used as the mobile phase at a ratio of 17:83 (*v*:*v*) and flow rate of 0.8 mL/min. Column temperature and detective wavelength were set as 30 °C and 250 nm, respectively. Qualitative and quantitative analysis of monosaccharides was based on comparison with retention times and integrated areas of the standards.

#### 3.4.2. Degree of Esterification

A total of 200 mg of the sample was dissolved in 20 mL H_2_O and stirred vigorously at 40 °C for 2 h. Then the cooled solution was titrated with NaOH (0.1 M) using phenolphthalein as an indicator and the volume of used NaOH was recorded as *V*_1_. The resulting solution was added to 10 mL of NaOH (0.1 M) and continued to be stirred at room temperature in the dark for another 2 h. Afterwards, 10 mL of HCl (0.1 M) was added and the pink color disappeared after complete mixing. The solution was again titrated with NaOH (0.1 M) and the used volume was recorded as *V*_2_. The degree of esterification (*DE*) was calculated as follows:

$$DE(\%)=\frac{V_{2}}{V_{1}+V_{2}}\times 100\%$$

#### 3.4.3. Infrared Spectroscopy Analysis

The infrared spectrum of the extract was analyzed using a Bruker VERTEX80V Fourier-transform infrared spectrometer in the wavelength range of 500–4000 cm^−1^ at a resolution of 2 cm^−1^. The sample was scanned 16 times.

### 3.5. In Vitro Skincare Effect Experiments 

#### 3.5.1. Moisturizing Test

Saturated ammonium sulfate solution was added on the bottom of a sealed desiccator. When a constant humidity was achieved, a glass plate covered with plastic tape was placed on the upper desiccator plate for 20 min and its weight was recorded as *m*. Then the polysaccharide extract solution (0.3%, 0.75% and 1.5% *w*/*v*) was evenly spread on the plastic tape (~2 mg/cm^2^) and the weight was immediately recorded as *m*_0_. The tape-covered glass plate carrying the extract solution was subsequently placed in the desiccator for 1 h, 2 h, 4 h, 8 h, 24 h and 48 h and the weight of entire glass plate (including tape and test sample) at each time point was recorded as *m_t_*. The moisturizing rate (*MR*) was calculated according to the following equation:

$$MR=\frac{m_{t}-m}{m_{0}-m}\times 100\%$$

For comparison, glycerol solution (5% *w*/*v*) and hyaluronic acid (HA) solution (0.5% *w*/*v*) were used as references.

#### 3.5.2. Total Phenolic Content Quantification

Total phenolic content (TPC) was determined by Folin–Ciocalteu assay, where 10 mg extract was dissolved in 1 mL H_2_O and subsequently mixed with 5 mL Folin–Ciocalteu regent (10% *v*/*v*) and 4 mL sodium carbonate solution (7.5% *w*/*v*). Then, the mixture was stirred in an ambient atmosphere in the dark for 1 h. The resulting solution was applied to a UV-vis spectrometer (UV-8000, Metash instrument Co., Ltd., Shanghai, China) measuring the absorbance at 765 nm. Quantification was based on the calibration curve of gallic acid treated under the same methods and the results were expressed as mg gallic acid equivalent (GAE) per gram of dry matter.

#### 3.5.3. DPPH-Free Radical Scavenging Activity

The anti-radical ability test of extract was determined according to the DPPH radical scavenging assay. Specifically, 1 mL of two-fold serial dilutions (8, 4, 2, 1, 0.5, 0.25 mg/mL) of the extract in ethanol (EtOH) was prepared and was mixed with 1 mL of 0.1 mM DPPH solution. The mixture was stirred at 25 °C for 30 min. A control sample was prepared using 1 mL pure H_2_O under the same protocol. For comparison, ascorbic acid dilutions with the same concentrations were used as references. All the solutions were applied to the UV-vis spectrometer to measure the absorbance at 517 nm and the anti-radical ability was expressed as the DPPH radical scavenging activity calculated using the following the formula:

$$DPPH\;radical\;scavenging\;activity\left(\%\right)=\left[1-\frac{A_{517nm}\left(sample\right)}{A_{517nm}\left(control\right)}\right]\times 100\%$$

#### 3.5.4. Hyaluronidase Inhibition Assay

Hyaluronidase (HAase) inhibition assay was performed by CAS Testing Technical Service (Guangzhou) Co., Ltd. (Guangzhou, China) based on a modified Elson–Morgan method [37]. Briefly, a 0.5 mL sample of polysaccharide extract solution (30 mg/mL) was preincubated with 0.5 mL of HAase (500 mU/mL) at 37 °C for 20 min. Subsequently, 0.1 mL of calcium chloride (2.5 M) was added and the mixture was incubated at 37 °C for an additional 20 min. The resulting solution was combined with 0.5 mL of hyaluronic acid potassium solution and allowed to react at 37 °C for 40 min. Upon addition of 0.5 mL acetylacetone solution and 0.5 mL sodium hydroxide solution, the mixture was heated in a boiling water bath for 15 min to stop the enzyme reaction. After cooling down to room temperature, the mixture was joined by 1 mL of *p*-dimethylaminobenzaldehyde (DMAB) reagent and reacted at 25 °C for 30 min until the color appears. The spectrometric absorbance at 530 nm was measured. The control experiments utilized dipotassium glycyrrhizate (DPG) as a positive control and sodium acetate buffer as a negative control (or blank) under exactly the same experimental conditions. HAase inhibition (*HI*) can be calculated following the formula:

$$HI=\left(1-\frac{C_{1}-D_{1}}{A_{1}-B_{1}}\right)\times 100\%$$

where *A*_1_ is the absorbance of solution with HAase and without sample; *B*_1_ represents the absorbance of the solution without HAase and sample; *C*_1_ stands for the absorbance of solution with HAase and sample; *D*_1_ means the absorbance of the solution with sample and without HAase.

#### 3.5.5. Elastase Inhibition Assay

Elastase inhibition (EI) assay was performed spectrophotometrically by CAS Testing Technical Service (Guangzhou) Co., Ltd., (Guangzhou, China) according to the method of Kraunsoe et al. [38]. In detail, 30 μL sample of polysaccharide extract solution (30 mg/mL) was diluted in 170 μL hydroxymethyl aminomethane hydrochloride buffer (Tris-HCl, 50 mM, pH = 8.0). After adding 50 μL elastase (600 mU/mL), the mixture was incubated at 25 °C for 15 min. Then 50 μL *N*-succ-(Ala)_3_-*p*-nitroanilide (AAA-pNA, 1.015 mM) solution was added, and the solution was vortexed and incubated at 25 °C for another 15 min. The resulting solution was applied to an RT-6100 microplate reader (Rayto Life and Analytical Science Co., Ltd., Shenzhen, China) to determine the absorbance at 410 nm. The performance of the assay was verified using epigallocatechin gallate (EGCG) as a positive control and H_2_O as negative control (or blank) under exactly the same experimental conditions. The *EI* was calculated according to the following equation:

$$EI=\left(1-\frac{C_{2}-D_{2}}{A_{2}-B_{2}}\right)\times 100\%$$

where *A*_2_ is the absorbance of solution with elastase and without sample; *B*_2_ represents the absorbance of solution without elastase and sample; *C*_2_ stands for the absorbance of solution with elastase and sample; *D*_2_ means the absorbance of solution with sample and without elastase.

## 4. Conclusions

In summary, the polysaccharide extract was obtained from the discarded *A. manihot* (L.) under the optimum UAE condition of solid–liquid ratio SL = 1:20, temperature T = 30 °C and time T = 40 min, achieving the extraction yield of 13.41%. Composition analysis reveals that Glc (44.65%), Rha (26.30%), GalA (12.50%) and Gal (9.86%) are the major monosaccharides of the extract. The extract shows a low DE value of 40.95%, and its FT−IR spectrum exhibits several characteristic peaks of polysaccharides. The moisture retention effect, TPC quantification, DPPH-free radical scavenging activity, anti-hyaluronidase and anti-elastase activities were tested to evaluate the in vitro skincare effect of the extract. Judging from the high 48 h moisture retention rate of 10.75%, the extract is believed as a good moisturizer. In addition, both the TPC value of 16.16 mg GAE/g (dw) and DPPH-free radical scavenging activity of 89.20% at the concentration of 2 mg/mL indicate the strong anti-oxidant properties of the extract. Furthermore, the anti-hyaluronidase activity and moderate anti-elastase activity were determined as 72.16% and 42.02%, respectively. In general, the in vitro skincare effect experiments suggest the moisturizing, anti-oxidant, anti-radical and anti-aging activities of the *A. manihot* root extract. This study opens a new avenue for the reuse of discarded *A. manihot* root to produce a potential high value-added cosmetic ingredient.

## 5. Patents

There are patents resulting from the work reported in this manuscript.

## Figures and Tables

**Table 1:** Proximate composition of *A. manihot* root ^1^.

	Moisture	Ash	Lipid	Protein	TDF	Carbohydrates
%dw	6.98 ± 0.21	18.34 ± 0.45	1.38 ± 0.11	10.31 ± 0.68	55.13 ± 0.03	7.86 ± 0.61

^1^ All the results are expressed as mean ± standard deviation of 3 replicates based on dry weight (1 g/100 g, %dw).

**Table 2:** Factors and levels of extraction conditions.

Entry	Factors		
	A. SL Ratio (g/mL)	B. Temperature (°C)	C. Time (min)
1	1:10	20	20
2	1:15	30	40
3	1:20	40	60

**Table 3:** The effects of different factors on extraction yields ^1^.

Entry	A. SL Ratio (g/mL)	B. Temperature (°C)	C. Time (min)	Yields (%) ^2^
1	1	1	1	6.99 ± 0.43
2	1	2	3	10.86 ± 0.55
3	1	3	2	9.68 ± 0.36
4	2	1	2	10.85 ± 0.68
5	2	2	1	12.42 ± 0.54
6	2	3	3	11.71 ± 1.00
7	3	1	3	10.94 ± 0.86
8	3	2	2	13.41 ± 0.24
9	3	3	1	12.04 ± 0.33
K_1_	9.18	9.59	10.48	
K_2_	11.66	12.23	11.31	
K_3_	12.13	11.14	11.17	
R	2.95	2.64	0.83	

^1^ K refers to the mean value for each level of each parameter and R is the range of K values of each parameter; ^2^ the extraction yields are expressed as mean ± standard deviation of 3 replicates.

**Table 4:** Hyaluronidase and elastase inhibition activity of the extract.

Material	Anti-Hyaluronidase (%) ^1^	Anti-Elastase (%) ^1^
*A. manihot* root extract	72.16 ± 1.79	42.02 ± 1.84
DPG	79.91 ± 1.49	NT ^2^
EGCG	NT ^2^	87.83 ± 1.59

^1^ The results are expressed as mean ± standard deviation of 3 replicates under the same concentration 0.1% (*w*/*v*). ^2^ NT = not tested.

## Data Availability

Data are contained within the article.
